# Supplementary material for: Quantum effects in an expanded Black–Scholes model
Source: Eur Phys J B. 2022 Aug 27;95(8):138. doi: 10.1140/epjb/s10051-022-00402-0 (PMC9419921; doi:10.1140/epjb/s10051-022-00402-0)
Supplement: Supplementary file 1 — (pdf 1946 KB) [file 10051_2022_402_MOESM1_ESM.pdf]

Supplementary material for:
Quantum effects in an expanded Black–Scholes model

Anantya Bhatnagar and Dimitri D. Vvedensky
The Blackett Laboratory, Imperial College London,
London SW7 2AZ, United Kingdom

(Dated: March 8, 2022)

Abstract

This document presents additional comparisons between actual European call option prices with calculated prices using the original Black–Scholes model and the expanded model suggested by Segal and Segal. Calculations have been carried out for stock options of 20 companies in different sectors of the S&P 500, each at three strike prices. These comparisons support the conclusions in the main manuscript by showing a wider range of price profiles and differences between the actual and calculated option prices.

I. INTRODUCTION

In the accompanying manuscript, comparisons are made between actual prices of European call options of stocks of several companies listed on the S&P 500 and prices calculated from the classical Black–Scholes model. These comparisons motivate an evaluation of the model proposed by Segal and Segal [1], who suggested the existence of additional processes that are not observable simultaneously with the processes governed by the Brownian motion in the Black–Scholes formulation. The pricing formulae obtained by Segal and Segal, in which the volatility is complex, were used to determine the imaginary part of the volatility, and thus to indicate the influence of the additional processes. In this document we provide additional comparisons between actual prices of European call options based on stocks of 20 S&P 500 companies and the original Black–Scholes model. The model of Segal and Segal is then used to account for the discrepancies between the market and Black–Scholes prices.

II. PRICING FORMULAE

The Black–Scholes pricing formulae [2–5] for a European call option are specified by five variables:

The strike price K

The current stock price S

The time T to expiration

The risk-free interest rate r

The volatility σ

The strike price and the time to expiration are set by the writer of the option. The current stock price is available from several sources to whatever level of resolution desired. We have used daily returns.

For the interest rate, we take the continuously compounded yield on a 3-month Treasury bill (T-bill) whose maturity date is closest to the expiry date of the option. T-bills are guaranteed by the government of the United States and are, therefore, considered to be free of default risk. Because the interest rates were so low, and the changes were essentially negligible over the 6-week period of our options window, we took the constant interest rate of 0.08% for all our calculations.

We determine the percentage variance σ for each stock from

$$\sigma = \beta \frac{\text{VIX}}{100}\,. \tag{1}$$

where VIX is the ticker symbol for the volatility index of the Chicago Board Options Exchange, which is a real-time measure of the volatility based on S&P 500 index options with near-term expiration dates. The factor β is a measure of the volatility of a stock compared to the volatility of all other stocks in a particular index, in our case, the S&P 500 index. The values of β [6] used for the calculations reported here are compiled in Table I.

For a constant risk-free interest rate and a time-dependent volatility, the Black–Scholes pricing formulae are [4]:

$$C^{(1)}(S,T) = SN(d_1^{(1)}) - Ke^{-rT}N(d_2^{(1)})\,, \tag{2}$$

where the new functions $d_1^{(1)}$ and $d_2^{(1)}$ are now given by

$$\begin{aligned} d_1^{(1)} &= \frac{\ln(S/K) + rT + \frac{1}{2}\int_0^T \sigma^2(\tau)\,d\tau}{\sqrt{\int_0^T \sigma^2(\tau)\,d\tau}}\,, \\ d_2^{(1)} &= \frac{\ln(S/K) + rT - \frac{1}{2}\int_0^T \sigma^2(\tau)\,d\tau}{\sqrt{\int_0^T \sigma^2(\tau)\,d\tau}}\,. \end{aligned} \tag{3}$$

Table I. Values of β used in (1) to calculate the volatility for the European call options in based on the stocks of the 20 companies from the S&P 500 whose prices are calculated from (2) and (3).

Ticker	Company Name	β	Ticker	Company Name	β
AAL	American Airlines Group	1.71	AAPL	Apple Inc.	1.36
AMD	Advanced Micro Devices, Inc.	2.32	AMZN	Amazon.com, Inc.	1.31
BA	Boeing Co.	1.41	BAC	Bank of America Corp.	1.57
BRK-B	Berkshire Hathaway Inc. Class B	0.84	C	Citigroup Inc.	1.82
GS	Goldman Sachs Group Inc,	1.42	INTC	Intel Corporation	0.68
JPM	JP Morgan Chase & Co.	1.12	M	Macy's Inc.	1.82
MAR	Marriott International Inc.	1.68	NFLX	Netflix Inc.	0.98
NKE	Nike Inc.	0.82	PFE	Pfizer Inc.	0.72
RCL	Royal Caribbean Cruises Ltd	2.76	TSLA	Tesla Inc.	1.97
WMT	Walmart Inc.	0.40	ZM	Zoom Video Communications Inc.	1.05

The corresponding pricing formulas obtained by Segal and Segal (modified for a time-dependent volatility) are [1]:

$$C^{(3)}(S,T) = S\exp\left\{\frac{1}{2}\int_0^T \left[\gamma^2(\tau)s^2(\tau) - k(\tau)\right] d\tau\right\} N(d_1^{(3)}) - Ke^{-rT}N(d_2^{(3)}), \tag{4}$$

with

$$\begin{aligned} d_1^{(3)} &= \frac{\ln(S/K) + rT + \int_0^T \left[\gamma^2(\tau)s^2(\tau) - \frac{1}{2}k(\tau)\right] d\tau}{\sqrt{\int_0^T \gamma^2(\tau)s^2(\tau)\,d\tau}}, \\ d_2^{(3)} &= \frac{\ln(S/K) + rT - \frac{1}{2}\int_0^T k(\tau)\,d\tau}{\sqrt{\int_0^T \gamma^2(\tau)s^2(\tau)\,d\tau}}, \end{aligned} \tag{5}$$

where the real part of $f(t)$ can be regarded as the process representing public information, whereas the imaginary part corresponds to a process that cannot be observed simultaneously with public information. Here, $s = |f(T)|$, $\gamma \geq 1$, and $k = s^2$ or $k = 0$ for Wiener or serial correlated processes, respectively.

III. COMPARISONS BETWEEN ACTUAL AND CALCULATED OPTION PRICES

The main result in the accompanying manuscript is that the imaginary part of the volatility in (4) and (5) can alleviate the differences between actual option prices and calculations based on (2) and (3) where the calculations underestimate the actual price. Our comparisons are based on options on 20 stocks from different sectors of the S&P 500. There are 11 sectors in the S&P 500. As of December 31, 2020 [7], the order of the sectors based on size, which is shown in parentheses, is as follows:

Information technology (27.6%)

Health care (13.5%)

Consumer discretionary (12.7%)

Communication services (10.8%)

Financials (10.4%)

Industrials (8.4%)

Consumer staples (6.5%)

Utilities (2.8%)

Materials (2.6%)

Real estate (2.4%)

Energy (2.3%).

The sectors of the 20 stocks whose option prices are calculated are shown on Table II. The following figures show the comparisons between the actual prices of European call options based on the stocks of the 20 S&P 500 companies in Tables I and II and the original Black–Scholes model based on (2) and (3), and between the expanded Black–Scholes model based

Table II. The sectors within the S&P 500 of the 20 stocks whose call option prices have been calculated.

Company Name	Sector
American Airlines Group	Industrials
Apple Inc.	Information Technology
Advanced Micro Devices Inc.	Information Technology
Amazon.com Inc.	Consumer Discretionary
Boeing Co.	Industrials
Bank of America Corp.	Financials
Berkshire Hathaway Inc. Class B	Financials
Citigroup Inc.	Financials
Goldman Sachs Group Inc.	Financials
Intel Corporation	Information Technology
JP Morgan Chase & Co.	Financials
Macy's Inc.	Consumer Discretionary
Marriott International Inc.	Consumer Discretionary
Netflix Inc.	Communication Services
Nike Inc.	Consumer Discretionary
Pfizer Inc.	Health Care
Royal Caribbean Cruises Ltd	Consumer Discretionary
Tesla Inc.	Consumer Discretionary
Walmart Inc.	Consumer Staples
Zoom Video Communications Inc.	Information Technology

on (4) and (5). In each figure, historical data [8] was used for the closing price of the call option on each trading day over the 6-week period from October 8, 2020 to November 20, 2020. Three strike prices are shown for each option.

---

[1] I. E. Segal, W. Segal, The Black-Scholes pricing formula in the quantum context, Proc. Natl. Acad. Sci. USA 95 (1998) 4072–4075. doi:https://doi.org/10.1073/pnas.95.7.4072.

[2] F. Black, M. Scholes, The pricing of options and corporate liabilities, J. Polit. Econ. 81 (1973) 637–654. doi:https://doi.org/10.1086/260062.

[3] R. Merton, Theory of rational option pricing, Bell J. Econ. 4 (1973) 141–183. doi:https://doi.org/10.2307/3003143.

[4] P. Wilmott, Paul Wilmott on Quantitative Finance, 2nd Edition, Vol. 1, Cambridge University Press, Cambridge, 2006.

[5] J. C. Hull, Options, Futures, and Other Derivatives, 9th Edition, Pearson, Boston, 2015.

[6] The Investopia Team, Using beta to understand a stock's risk, Investopedia at https://www.investopedia.com/investing/beta-gauging-price-fluctuations/.

[7] https://www.etf.com/sections/etf-strategist-corner/sector-sector-sp-500?nopaging=1.

[8] https://www.bloomberg.com/markets/stocks.

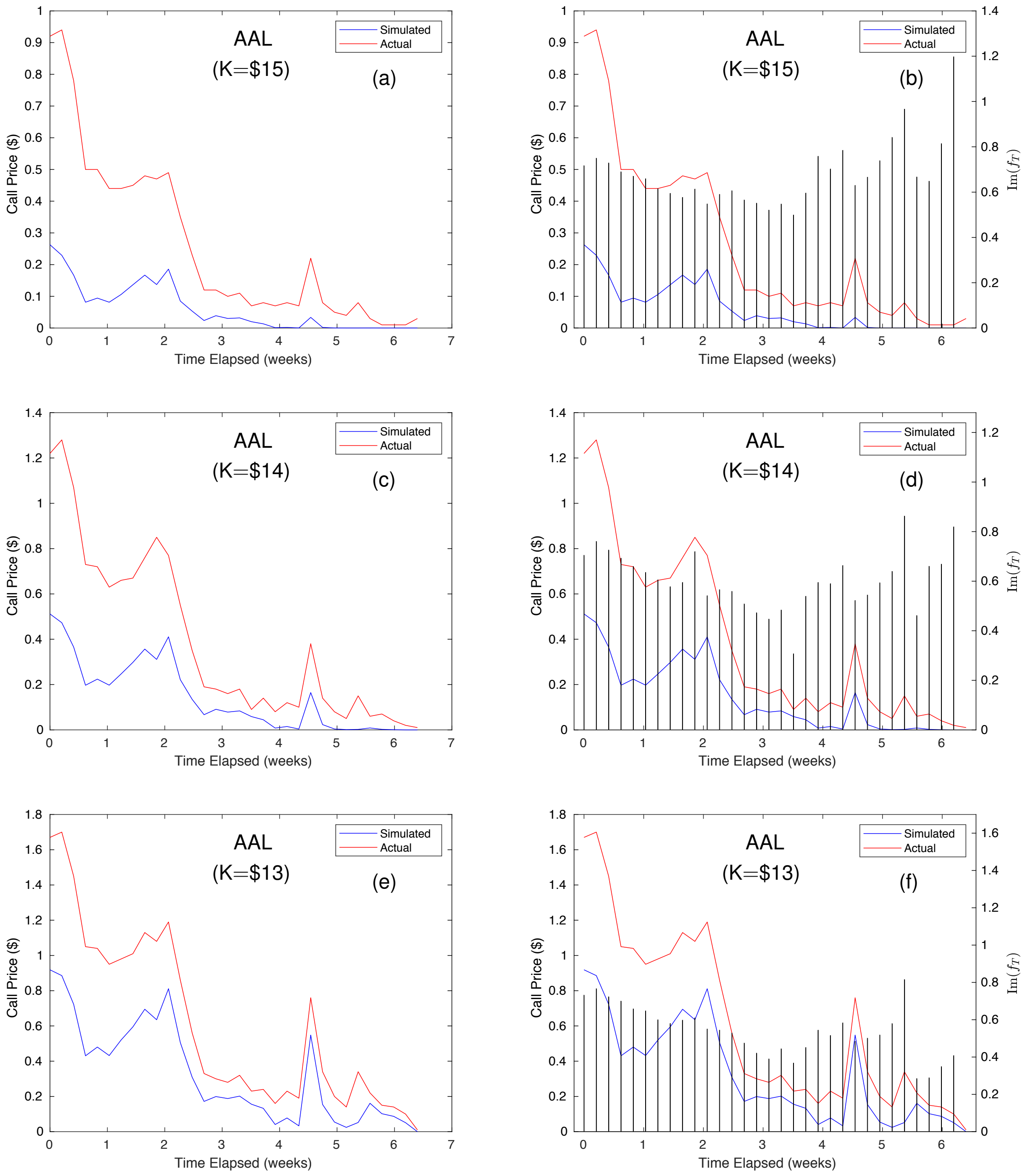

Figure 1. Comparison between actual prices of a European call option (red lines) for stocks of the American Airlines Group and (a,c,e) the original Black–Scholes model based on the pricing formulae (2) and (3) (blue lines), and (b,c,d) the expanded Black–Scholes model based on the pricing formulae (4) and (5) of the expanded Black–Scholes model proposed by Segal and Segal (black vertical lines).

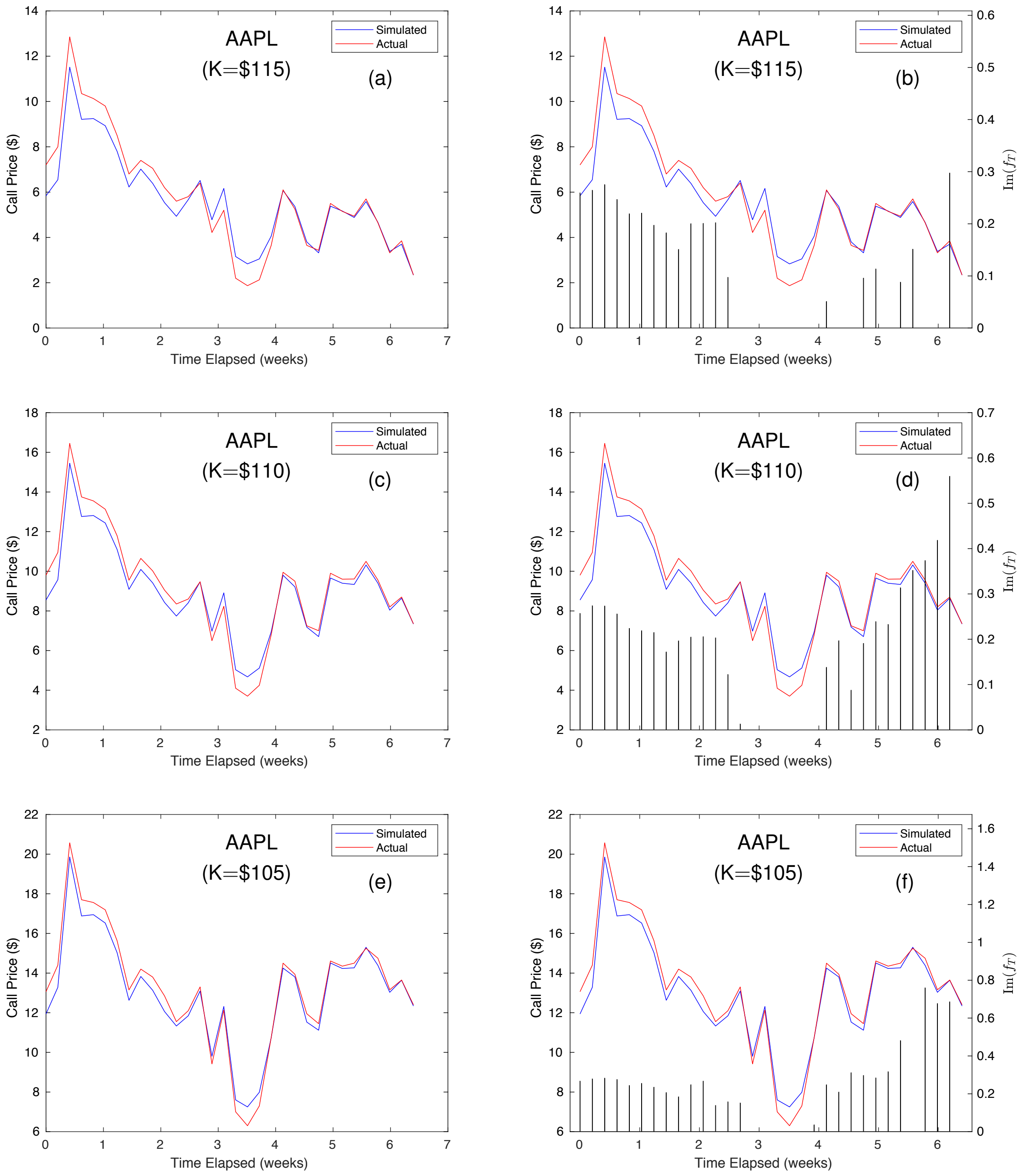

Figure 2. Comparison between actual prices of a European call option (red lines) for stocks of Apple Inc. and (a,c,e) the original Black–Scholes model based on the pricing formulae (2) and (3) (blue lines), and (b,c,d) the expanded Black–Scholes model based on the pricing formulae (4) and (5) of the expanded Black–Scholes model proposed by Segal and Segal (black vertical lines).

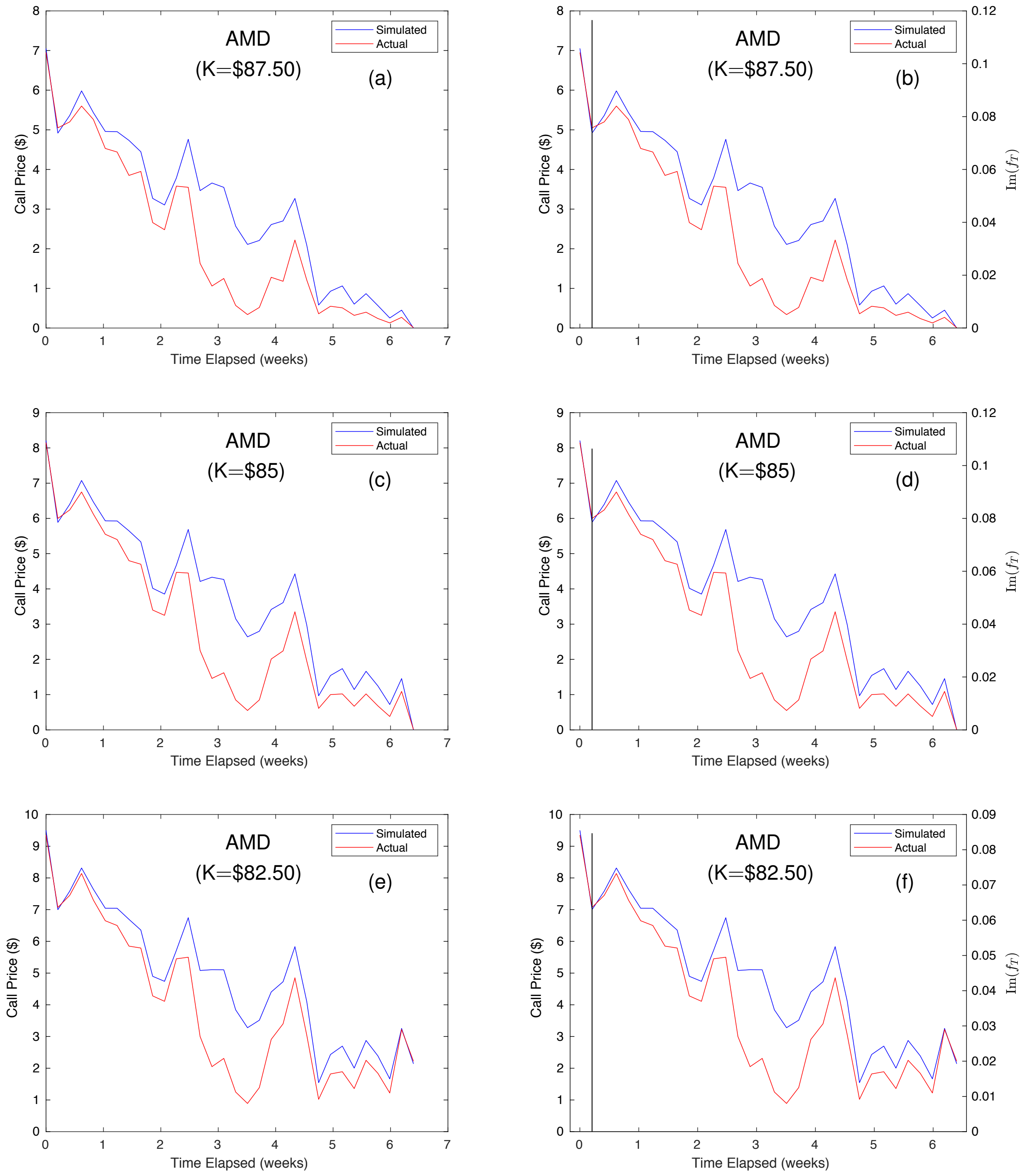

Figure 3. Comparison between actual prices of a European call option (red lines) for stocks of Advanced Micro Devices Inc. and (a,c,e) the original Black–Scholes model based on the pricing formulae (2) and (3) (blue lines), and (b,c,d) the expanded Black–Scholes model based on the pricing formulae (4) and (5) of the expanded Black–Scholes model proposed by Segal and Segal (black vertical lines).

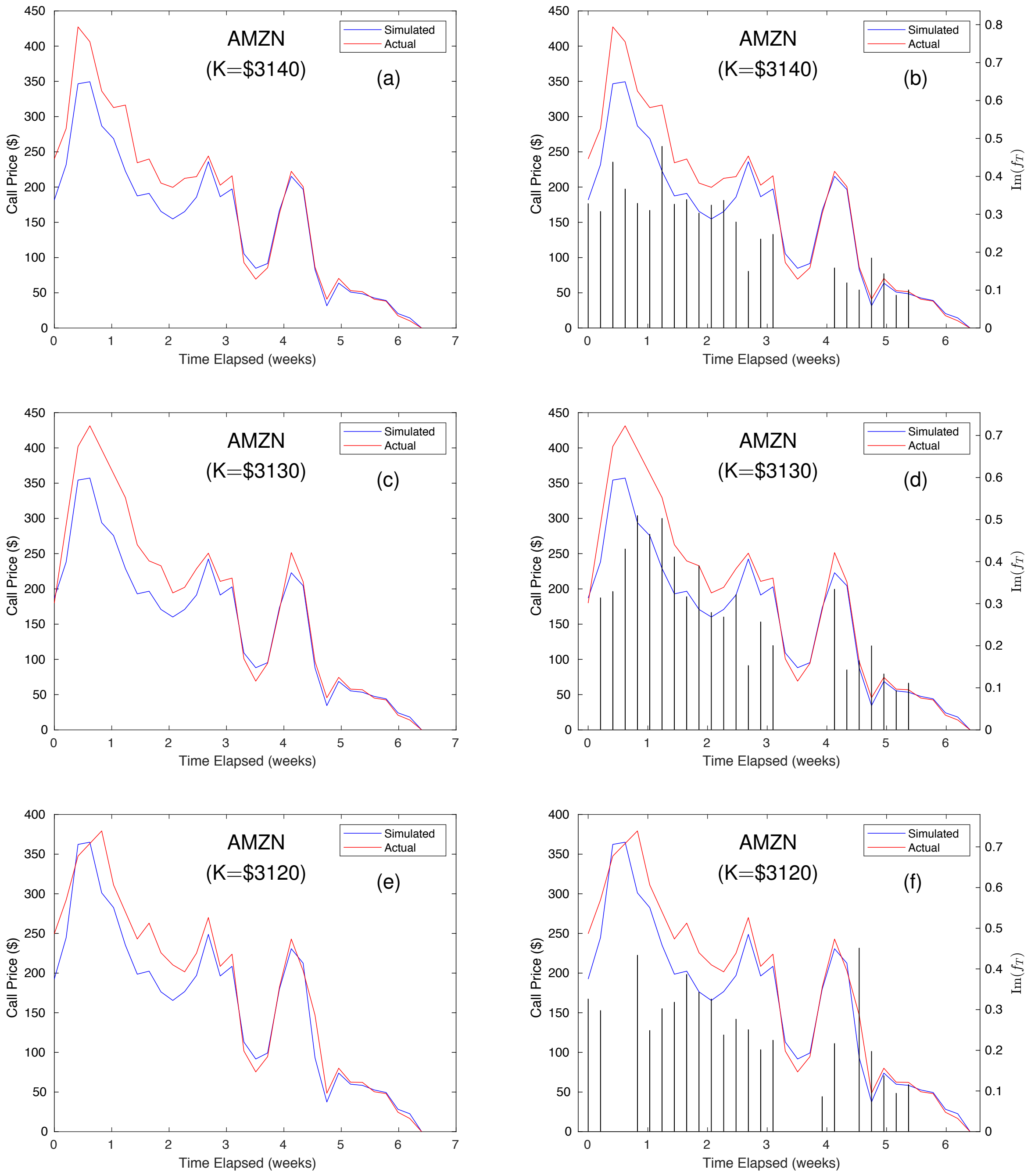

Figure 4. Comparison between actual prices of a European call option (red lines) for stocks of Amazon.com Inc. and (a,c,e) the original Black–Scholes model based on the pricing formulae (2) and (3) (blue lines), and (b,c,d) the expanded Black–Scholes model based on the pricing formulae (4) and (5) of the expanded Black–Scholes model proposed by Segal and Segal (black vertical lines).

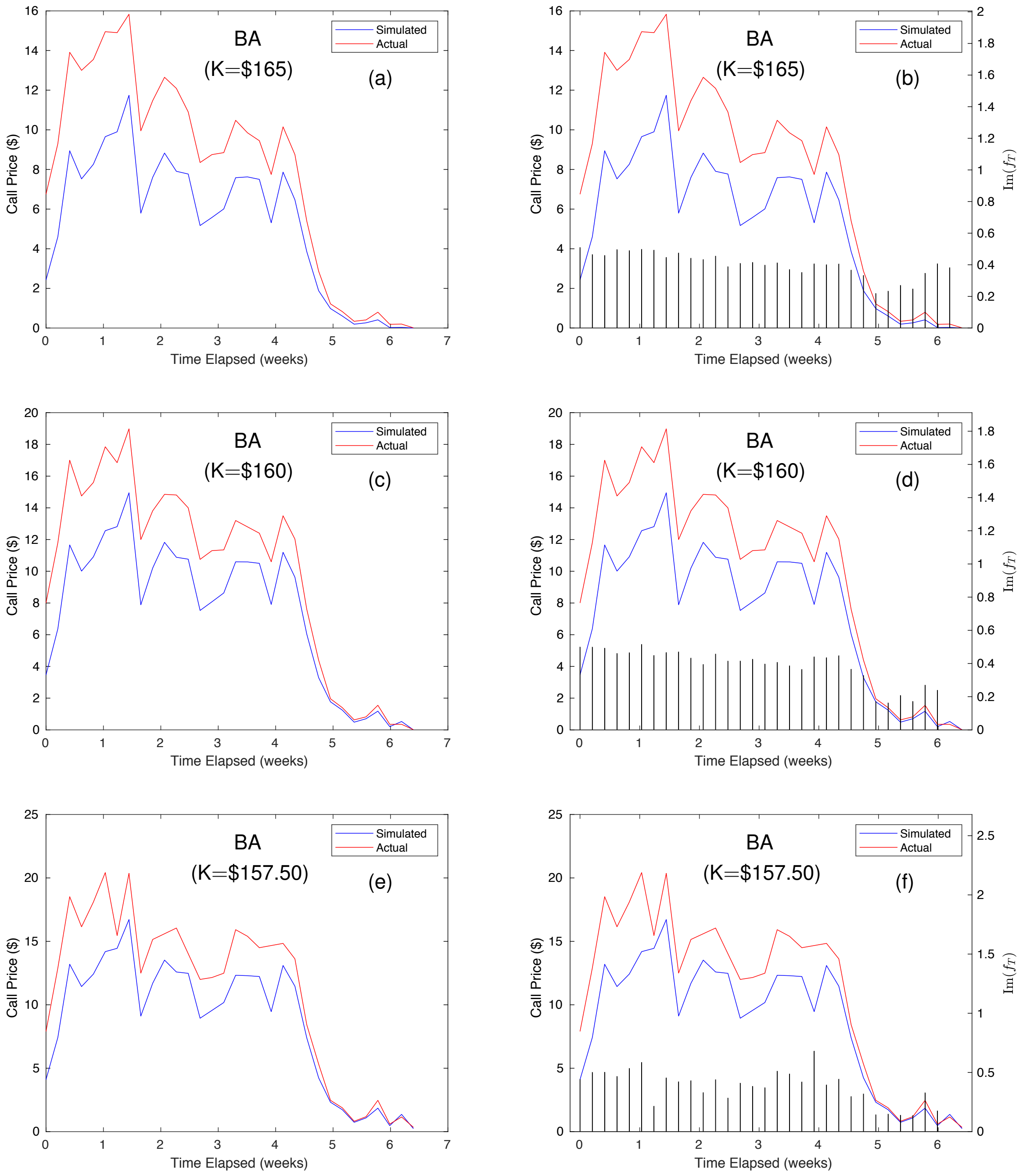

Figure 5. Comparison between actual prices of a European call option (red lines) for stocks of the Boeing Co. and (a,c,e) the original Black–Scholes model based on the pricing formulae (2) and (3) (blue lines), and (b,c,d) the expanded Black–Scholes model based on the pricing formulae (4) and (5) of the expanded Black–Scholes model proposed by Segal and Segal (black vertical lines).

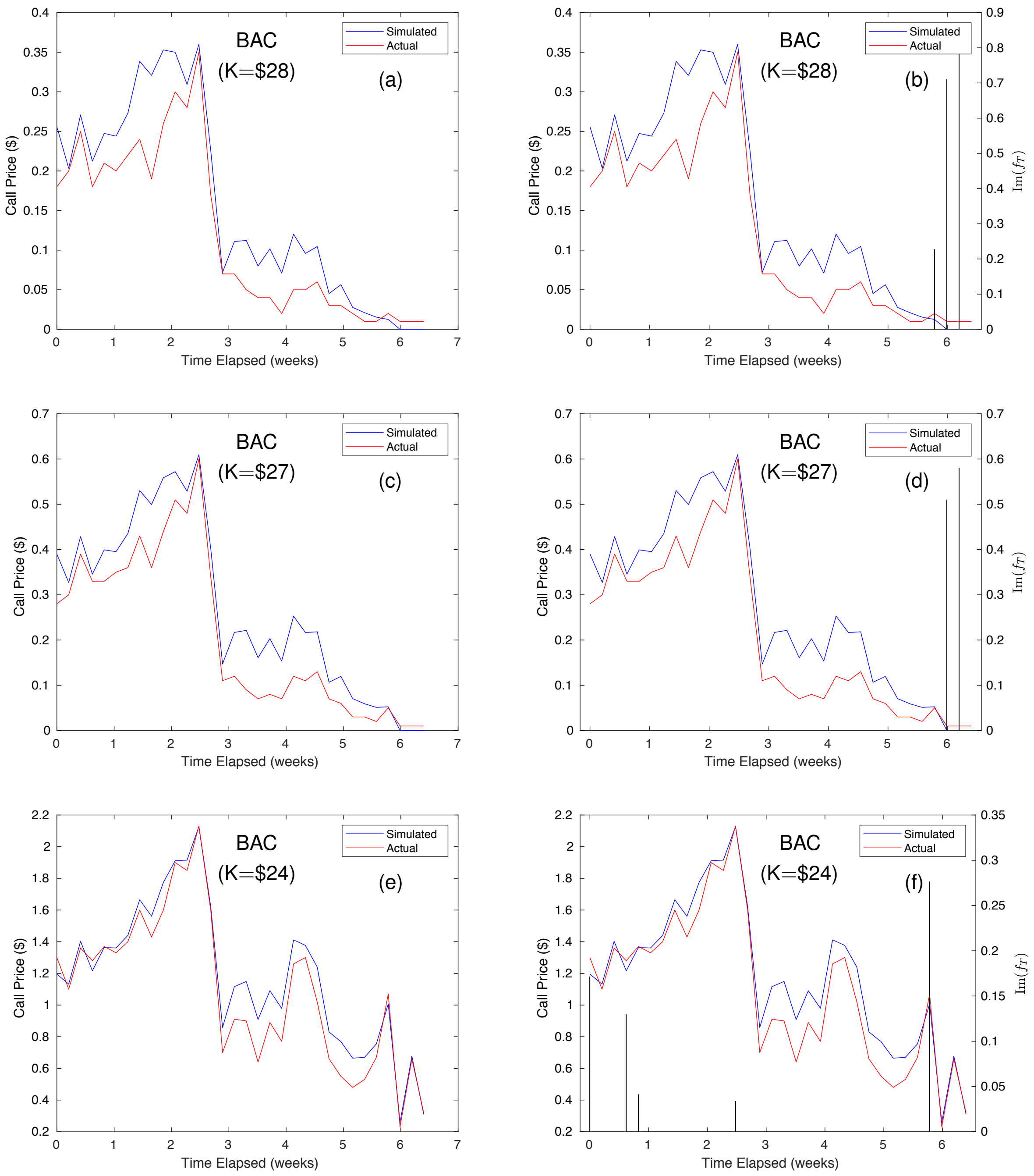

Figure 6. Comparison between actual prices of a European call option (red lines) for stocks of the Bank of America Corp. and (a,c,e) the original Black–Scholes model based on the pricing formulae (2) and (3) (blue lines), and (b,c,d) the expanded Black–Scholes model based on the pricing formulae (4) and (5) of the expanded Black–Scholes model proposed by Segal and Segal (black vertical lines).

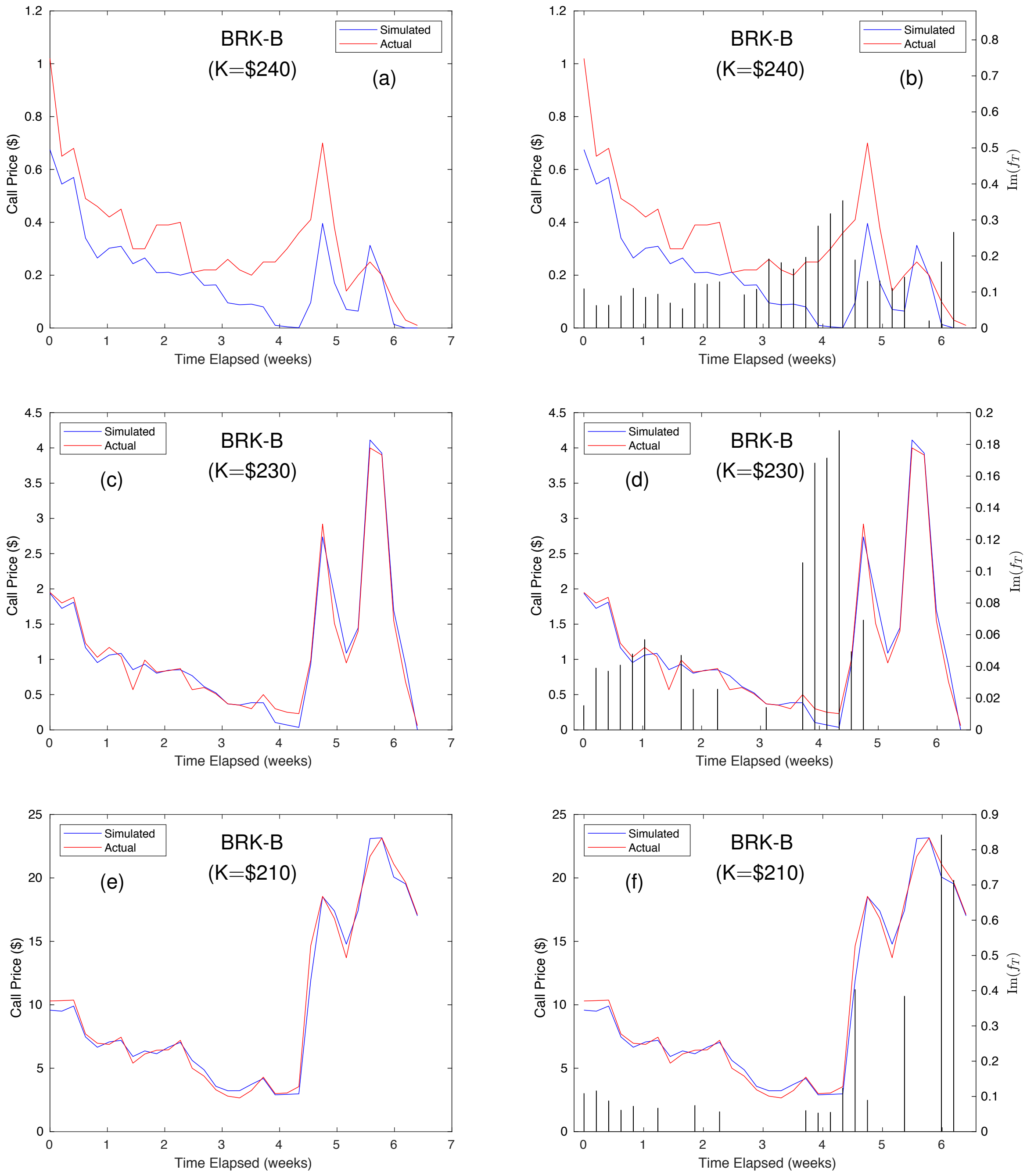

Figure 7. Comparison between actual prices of a European call option (red lines) for stocks of Berkshire Hathaway Inc. Class B and (a,c,e) the original Black–Scholes model based on the pricing formulae (2) and (3) (blue lines), and (b,c,d) the expanded Black–Scholes model based on the pricing formulae (4) and (5) of the expanded Black–Scholes model proposed by Segal and Segal (black vertical lines).

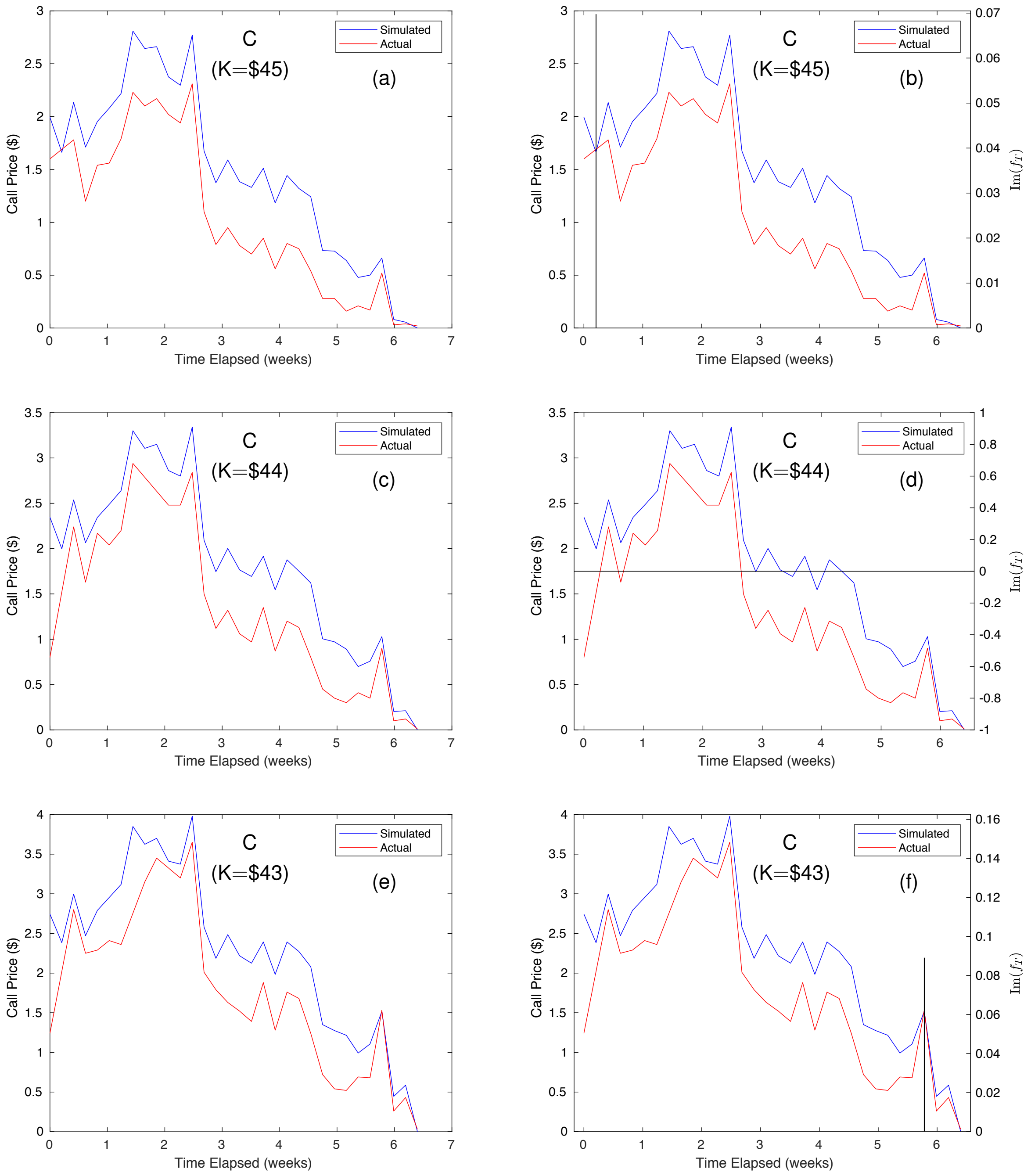

Figure 8. Comparison between actual prices of a European call option (red lines) for stocks of Citigroup Inc. Class B and (a,c,e) the original Black–Scholes model based on the pricing formulae (2) and (3) (blue lines), and (b,c,d) the expanded Black–Scholes model based on the pricing formulae (4) and (5) of the expanded Black–Scholes model proposed by Segal and Segal (black vertical lines).

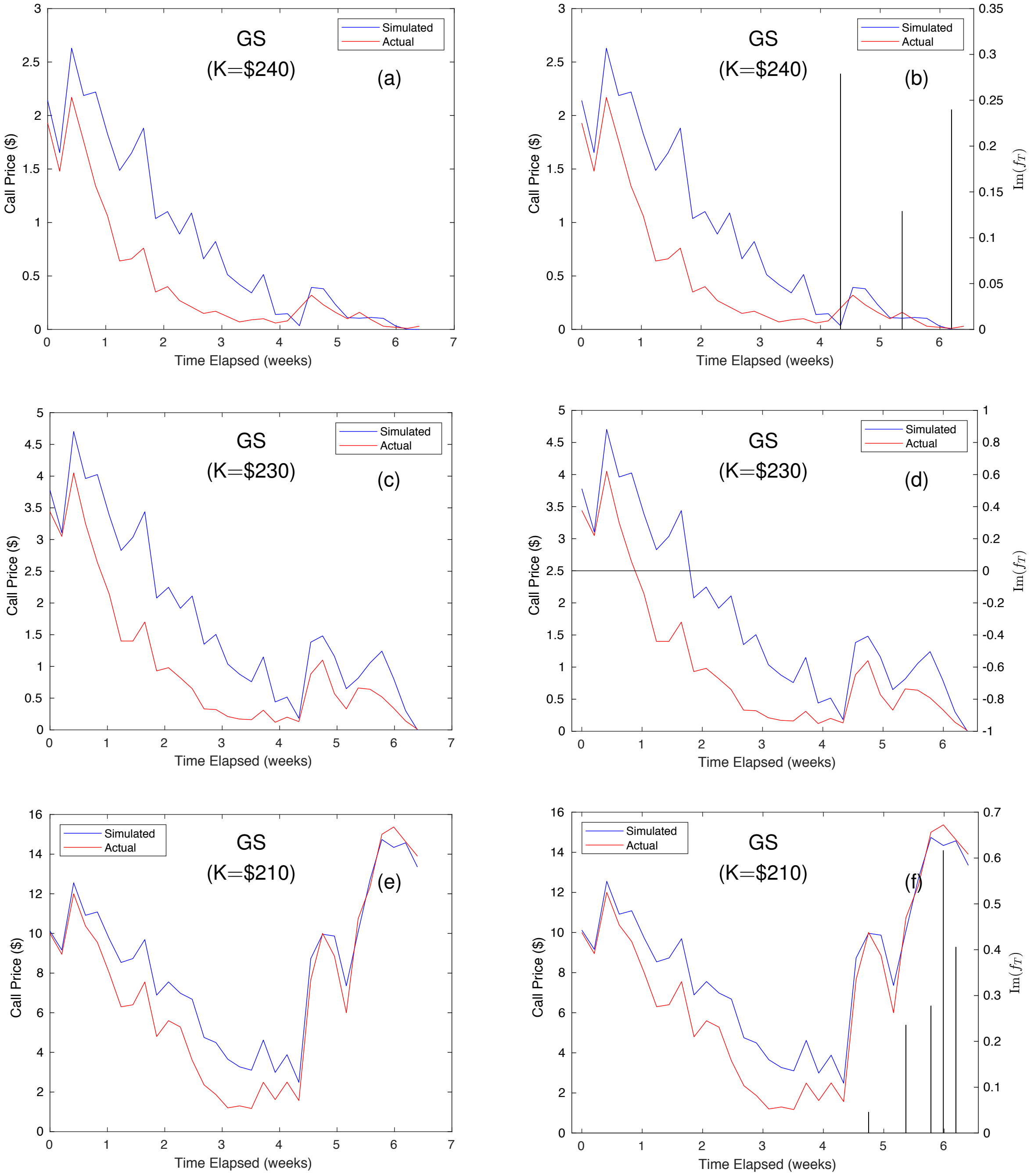

Figure 9. Comparison between actual prices of a European call option (red lines) for stocks of the Goldman Sachs Group Inc. and (a,c,e) the original Black–Scholes model based on the pricing formulae (2) and (3) (blue lines), and (b,c,d) the expanded Black–Scholes model based on the pricing formulae (4) and (5) of the expanded Black–Scholes model proposed by Segal and Segal (black vertical lines).

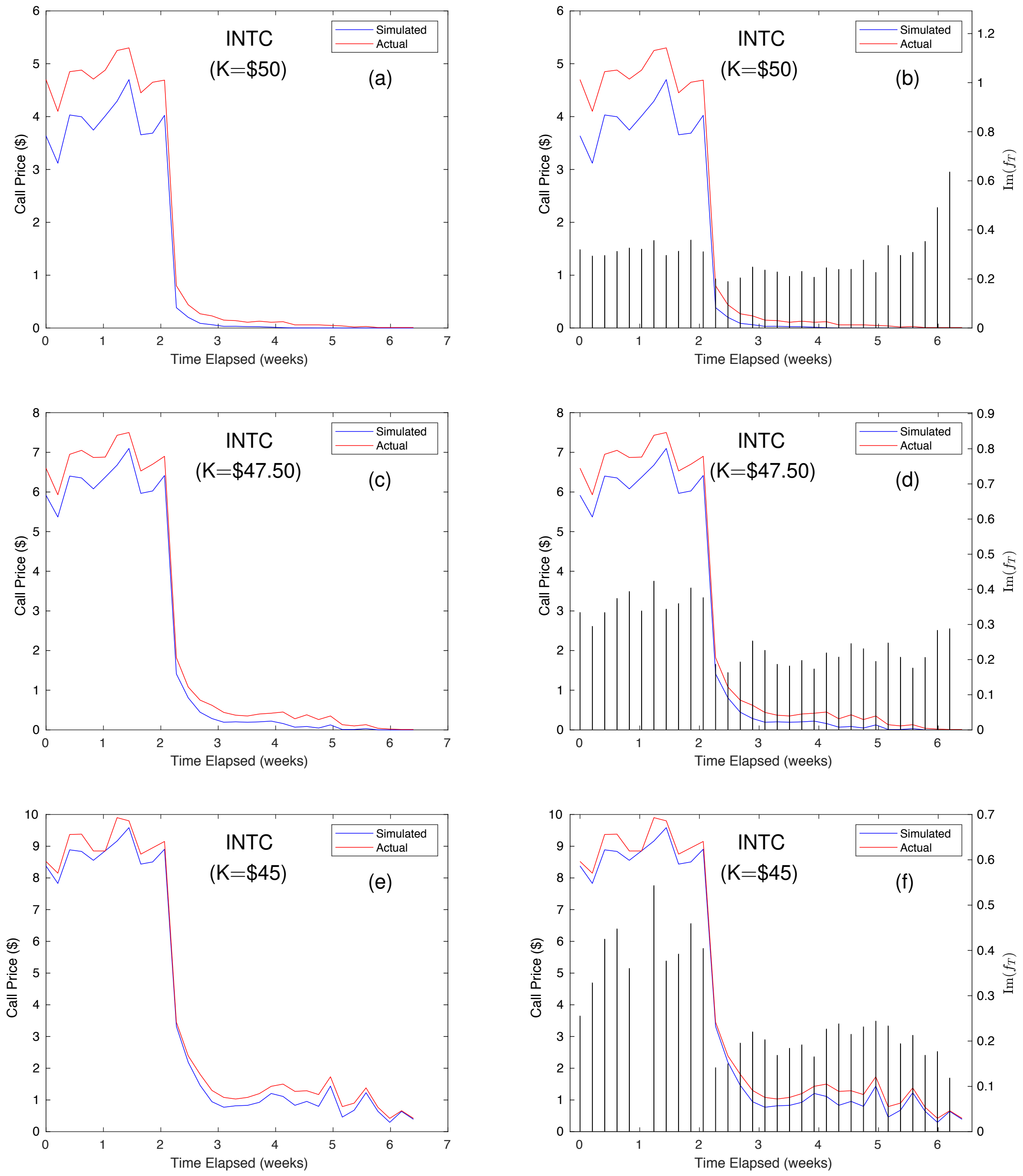

Figure 10. Comparison between actual prices of a European call option (red lines) for stocks of the Intel Corporation and (a,c,e) the original Black–Scholes model based on the pricing formulae (2) and (3) (blue lines), and (b,c,d) the expanded Black–Scholes model based on the pricing formulae (4) and (5) of the expanded Black–Scholes model proposed by Segal and Segal (black vertical lines).

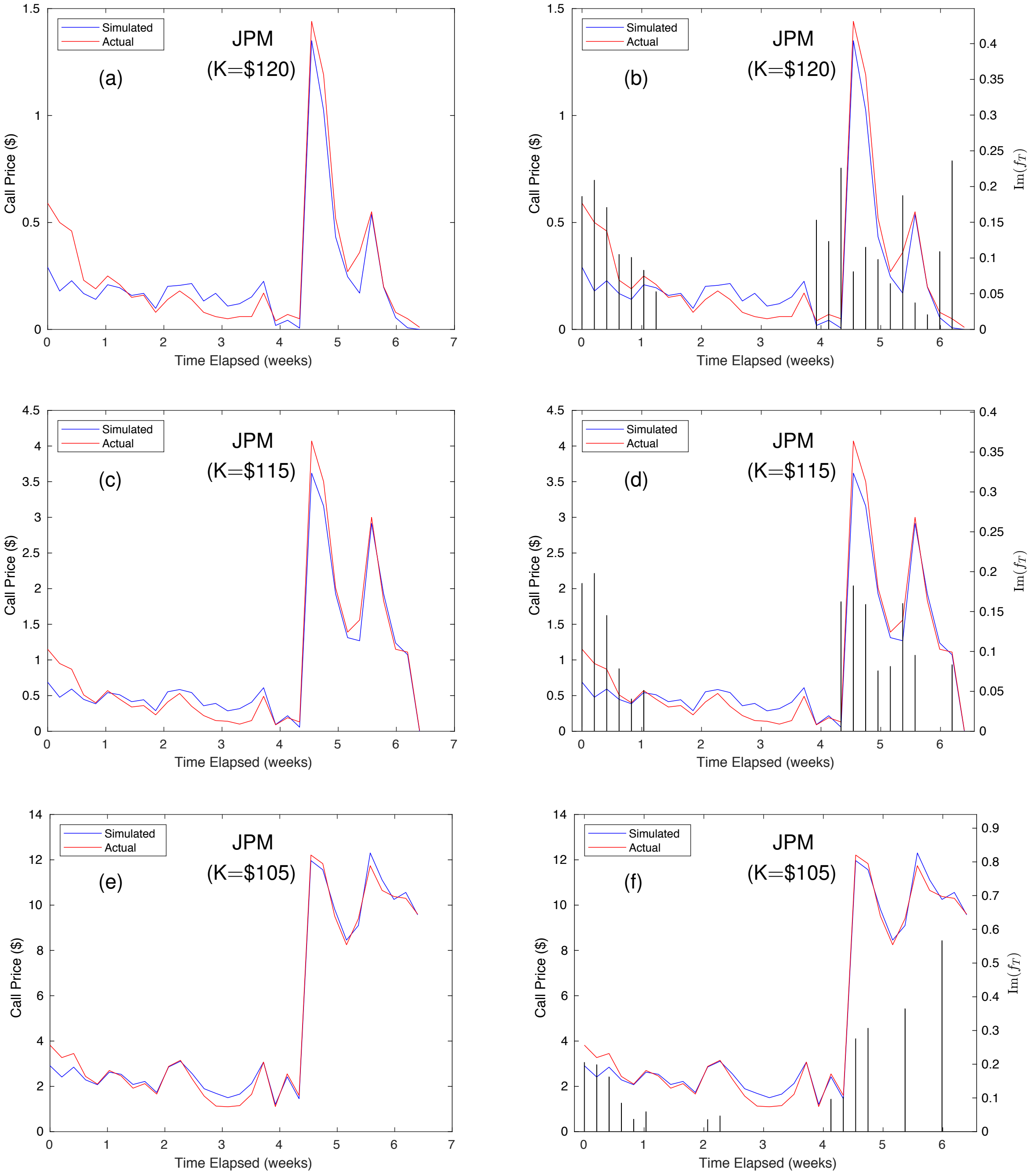

Figure 11. Comparison between actual prices of a European call option (red lines) for stocks of JP Morgan Chase & Co. and (a,c,e) the original Black–Scholes model based on the pricing formulae (2) and (3) (blue lines), and (b,c,d) the expanded Black–Scholes model based on the pricing formulae (4) and (5) of the expanded Black–Scholes model proposed by Segal and Segal (black vertical lines).

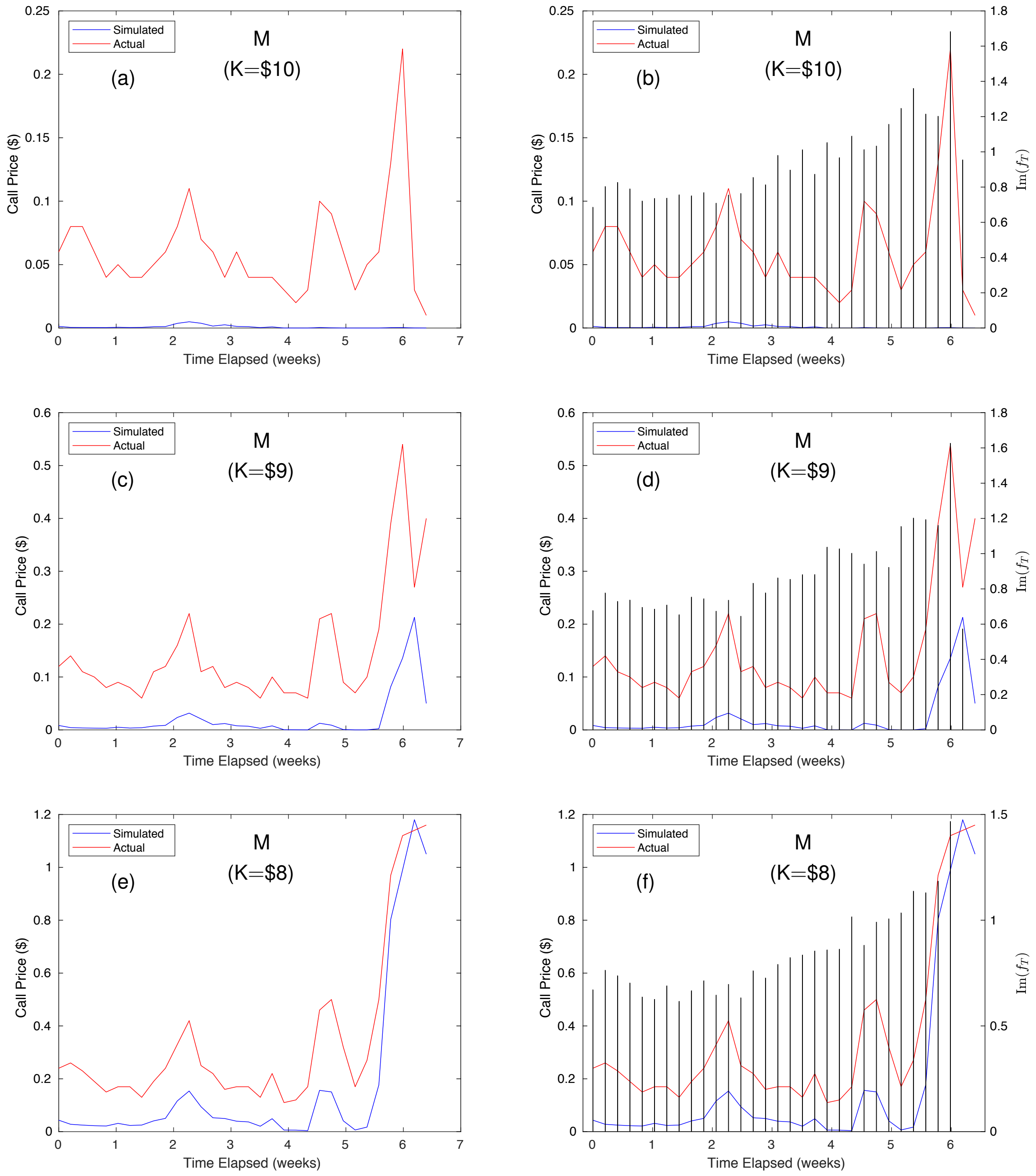

Figure 12. Comparison between actual prices of a European call option (red lines) for stocks of Macy's Inc. and (a,c,e) the original Black–Scholes model based on the pricing formulae (2) and (3) (blue lines), and (b,c,d) the expanded Black–Scholes model based on the pricing formulae (4) and (5) of the expanded Black–Scholes model proposed by Segal and Segal (black vertical lines).

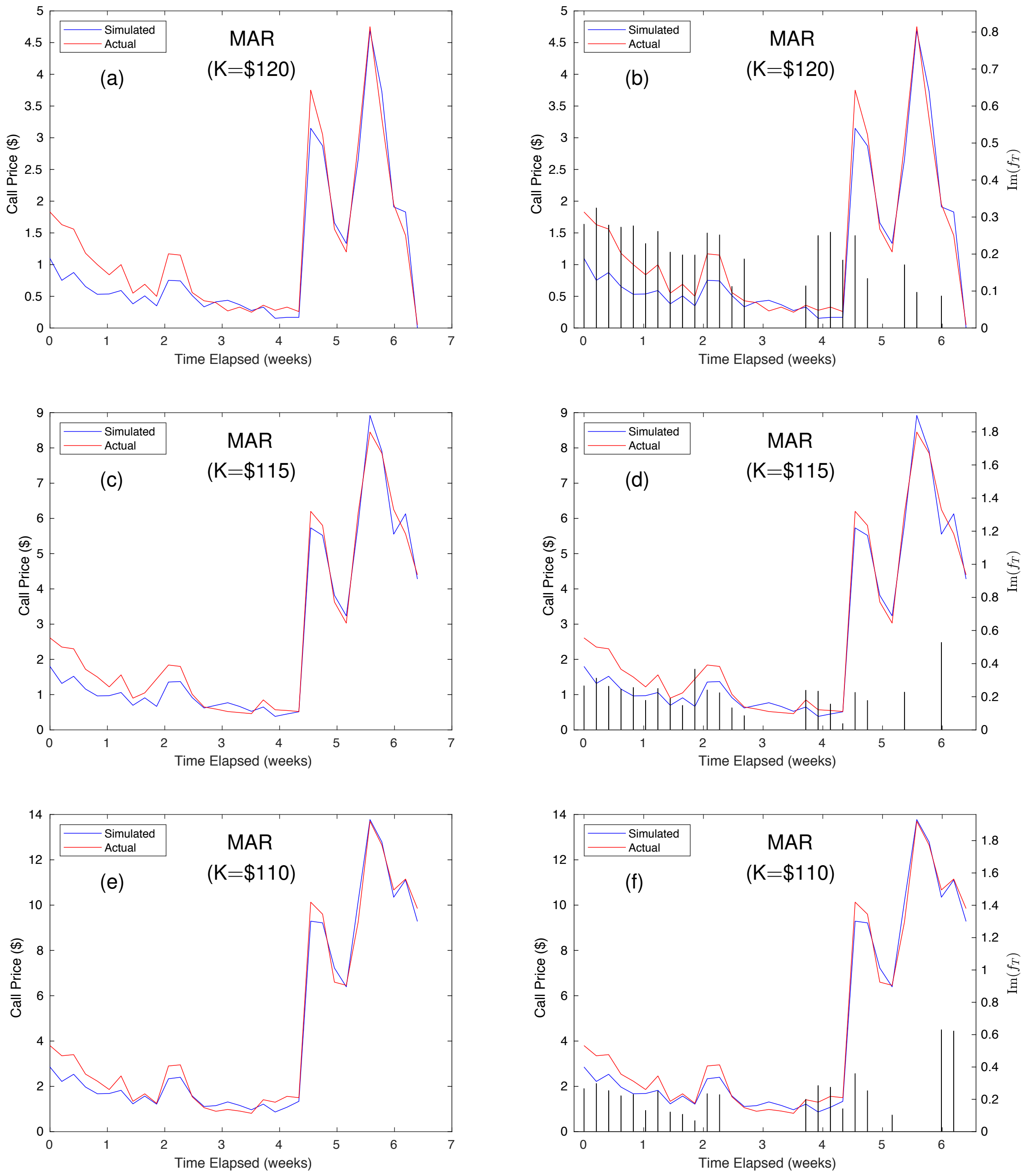

Figure 13. Comparison between actual prices of a European call option (red lines) for stocks of Marriott International Inc. and (a,c,e) the original Black–Scholes model based on the pricing formulae (2) and (3) (blue lines), and (b,c,d) the expanded Black–Scholes model based on the pricing formulae (4) and (5) of the expanded Black–Scholes model proposed by Segal and Segal (black vertical lines).

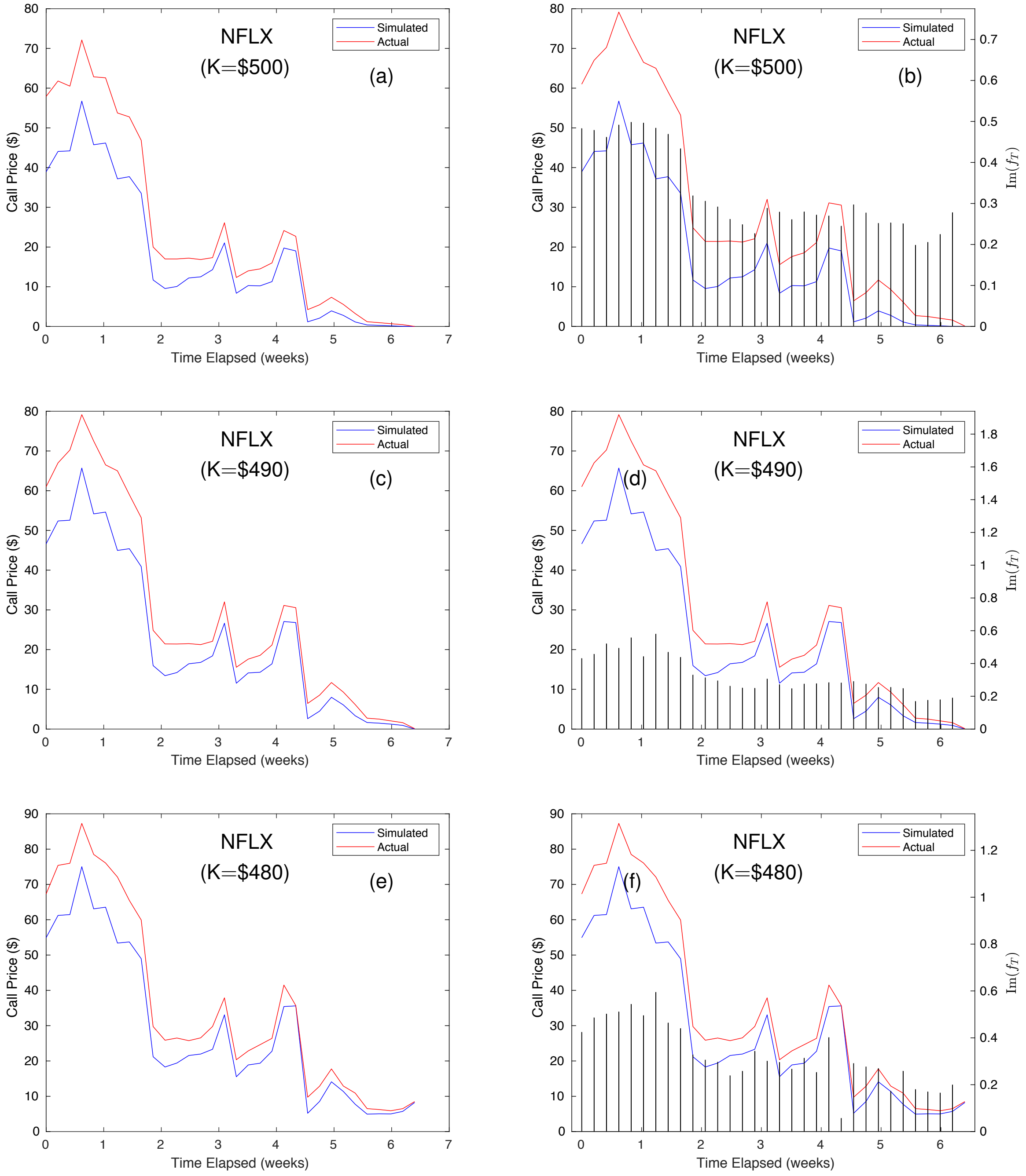

Figure 14. Comparison between actual prices of a European call option (red lines) for stocks of Netflix Inc. and (a,c,e) the original Black–Scholes model based on the pricing formulae (2) and (3) (blue lines), and (b,c,d) the expanded Black–Scholes model based on the pricing formulae (4) and (5) of the expanded Black–Scholes model proposed by Segal and Segal (black vertical lines).

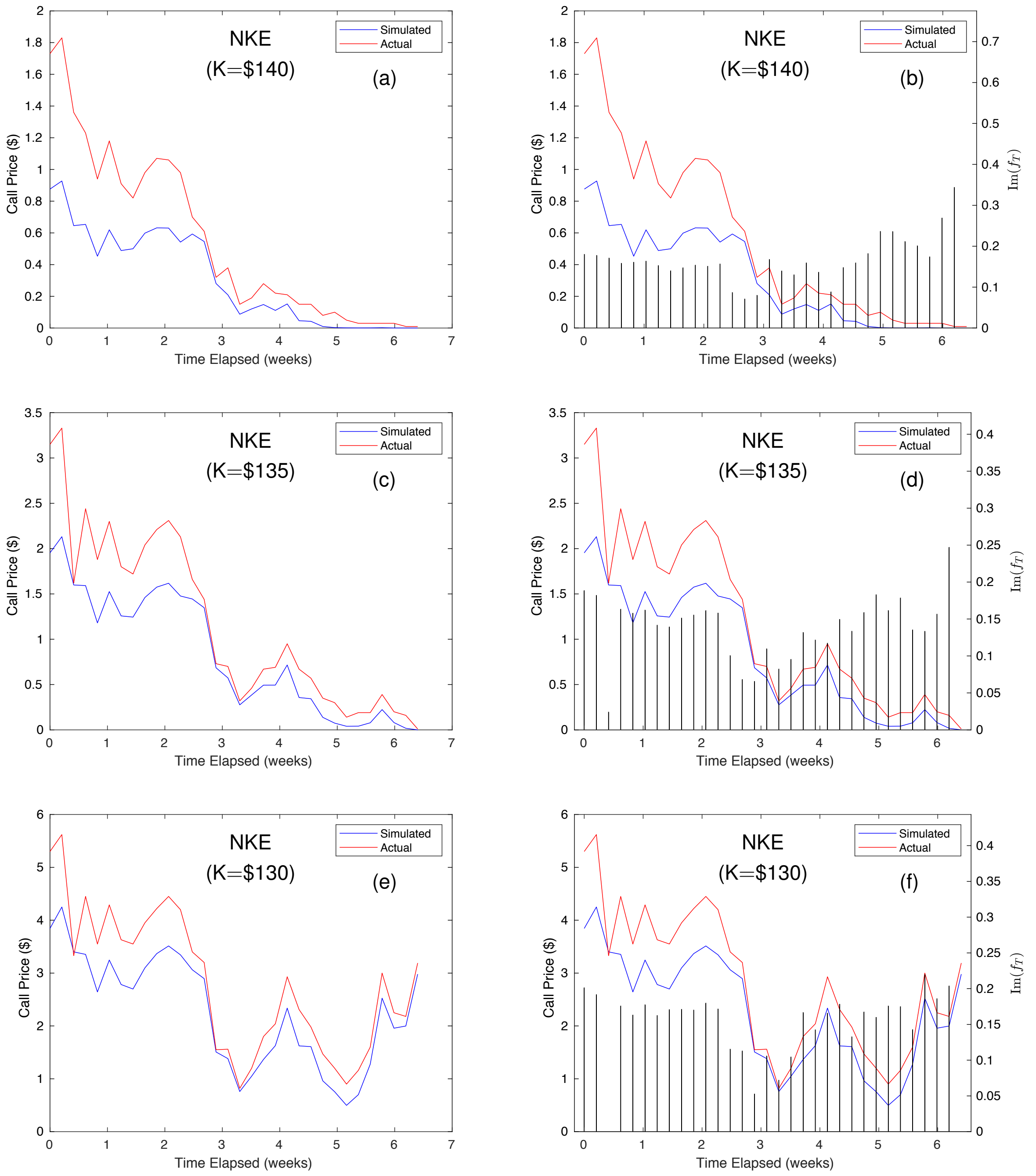

Figure 15. Comparison between actual prices of a European call option (red lines) for stocks of Nike Inc. and (a,c,e) the original Black–Scholes model based on the pricing formulae (2) and (3) (blue lines), and (b,c,d) the expanded Black–Scholes model based on the pricing formulae (4) and (5) of the expanded Black–Scholes model proposed by Segal and Segal (black vertical lines).

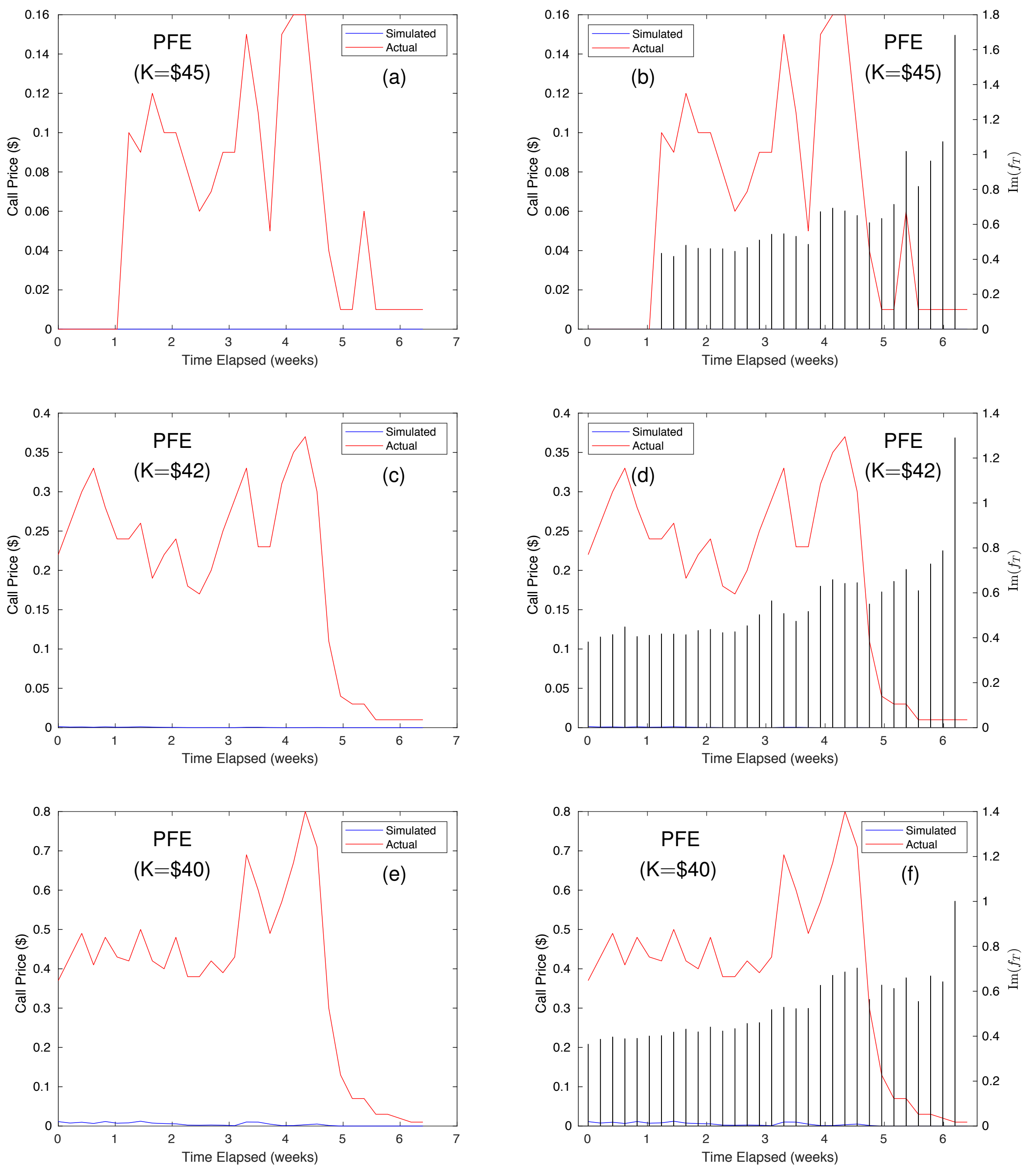

Figure 16. Comparison between actual prices of a European call option (red lines) for stocks of Pfizer Inc. and (a,c,e) the original Black–Scholes model based on the pricing formulae (2) and (3) (blue lines), and (b,c,d) the expanded Black–Scholes model based on the pricing formulae (4) and (5) of the expanded Black–Scholes model proposed by Segal and Segal (black vertical lines).

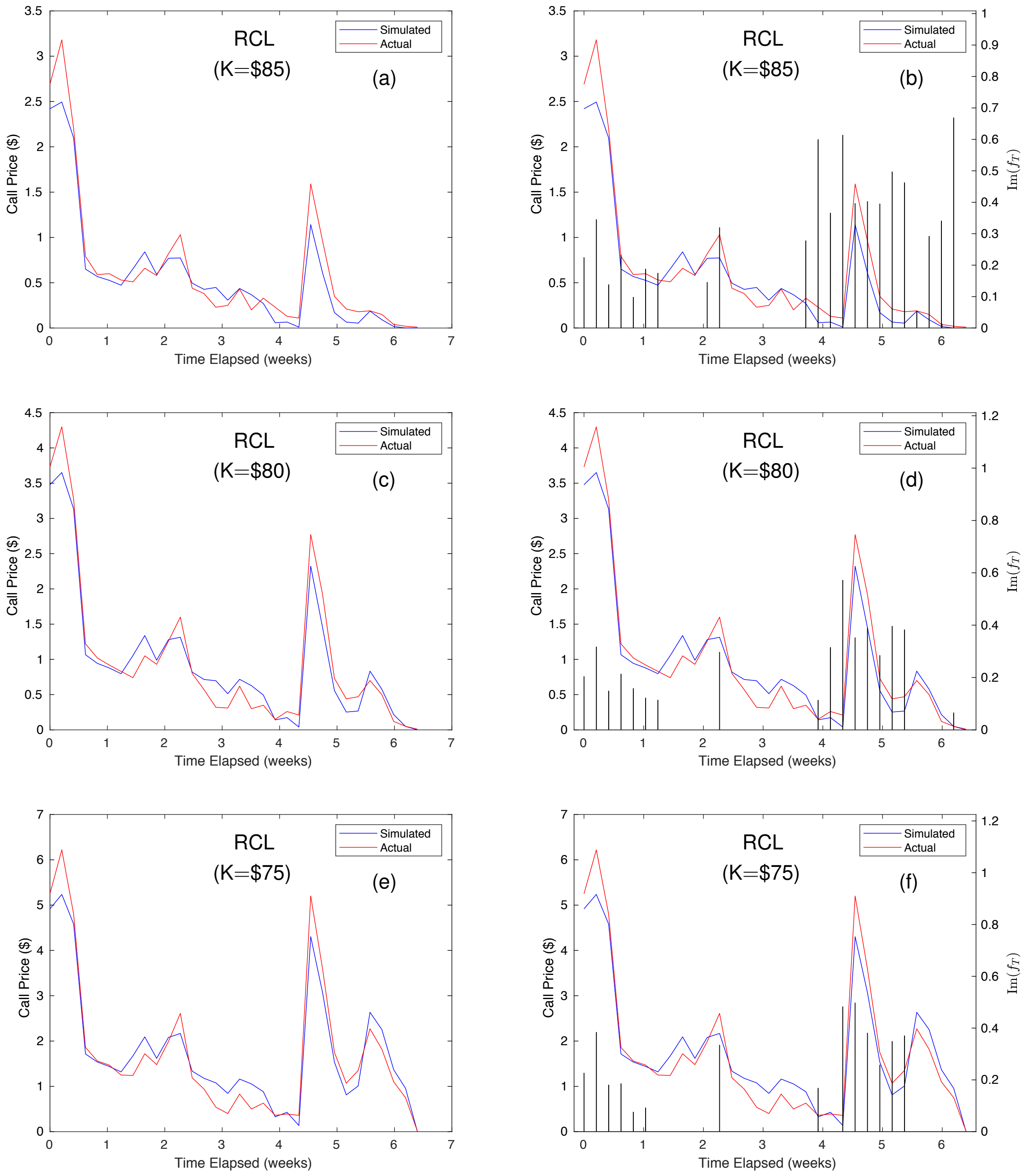

Figure 17. Comparison between actual prices of a European call option (red lines) for stocks of Royal Caribbean Cruises Ltd and (a,c,e) the original Black–Scholes model based on the pricing formulae (2) and (3) (blue lines), and (b,c,d) the expanded Black–Scholes model based on the pricing formulae (4) and (5) of the expanded Black–Scholes model proposed by Segal and Segal (black vertical lines).

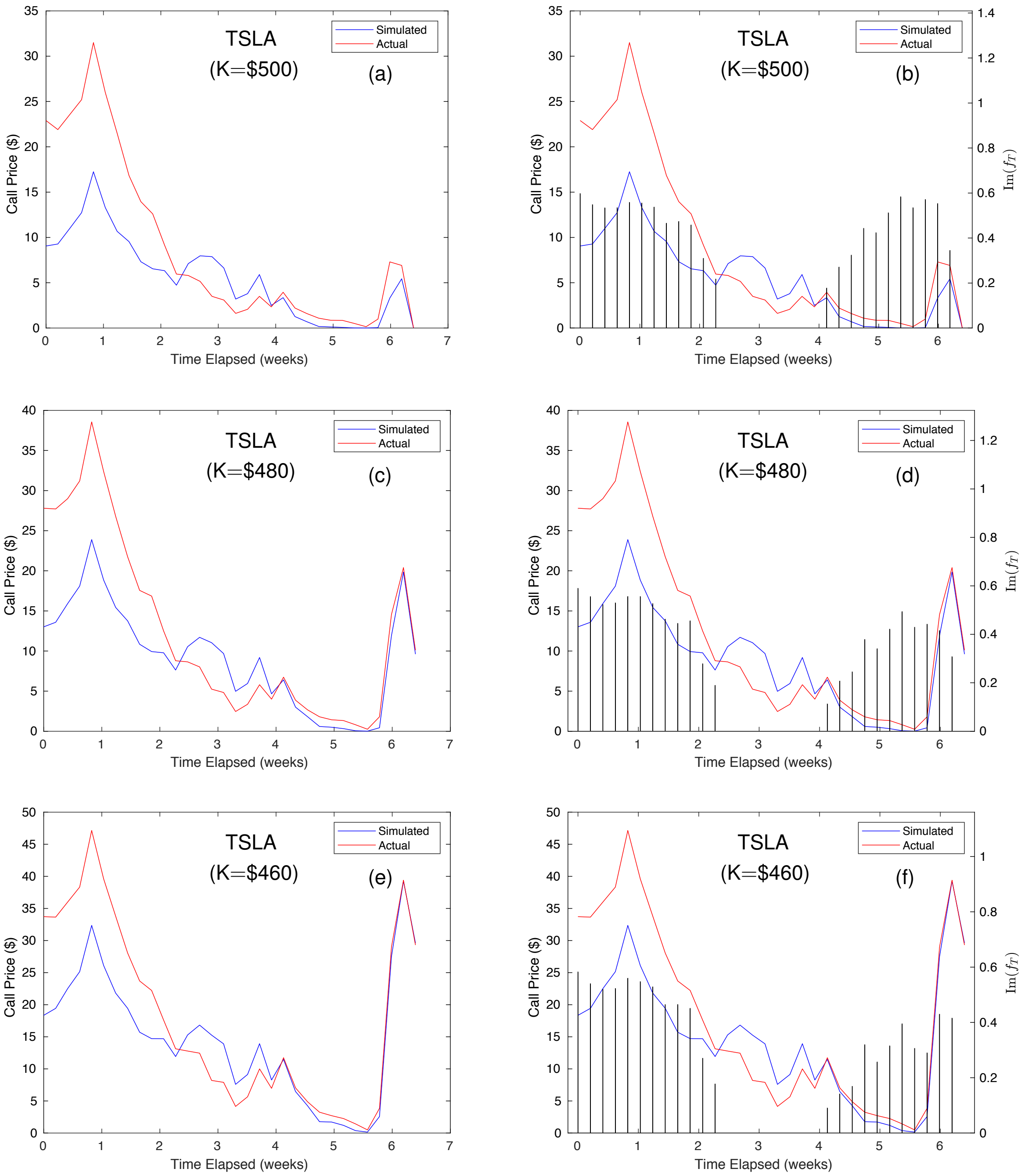

Figure 18. Comparison between actual prices of a European call option (red lines) for stocks of Tesla Inc. and (a,c,e) the original Black–Scholes model based on the pricing formulae (2) and (3) (blue lines), and (b,c,d) the expanded Black–Scholes model based on the pricing formulae (4) and (5) of the expanded Black–Scholes model proposed by Segal and Segal (black vertical lines).

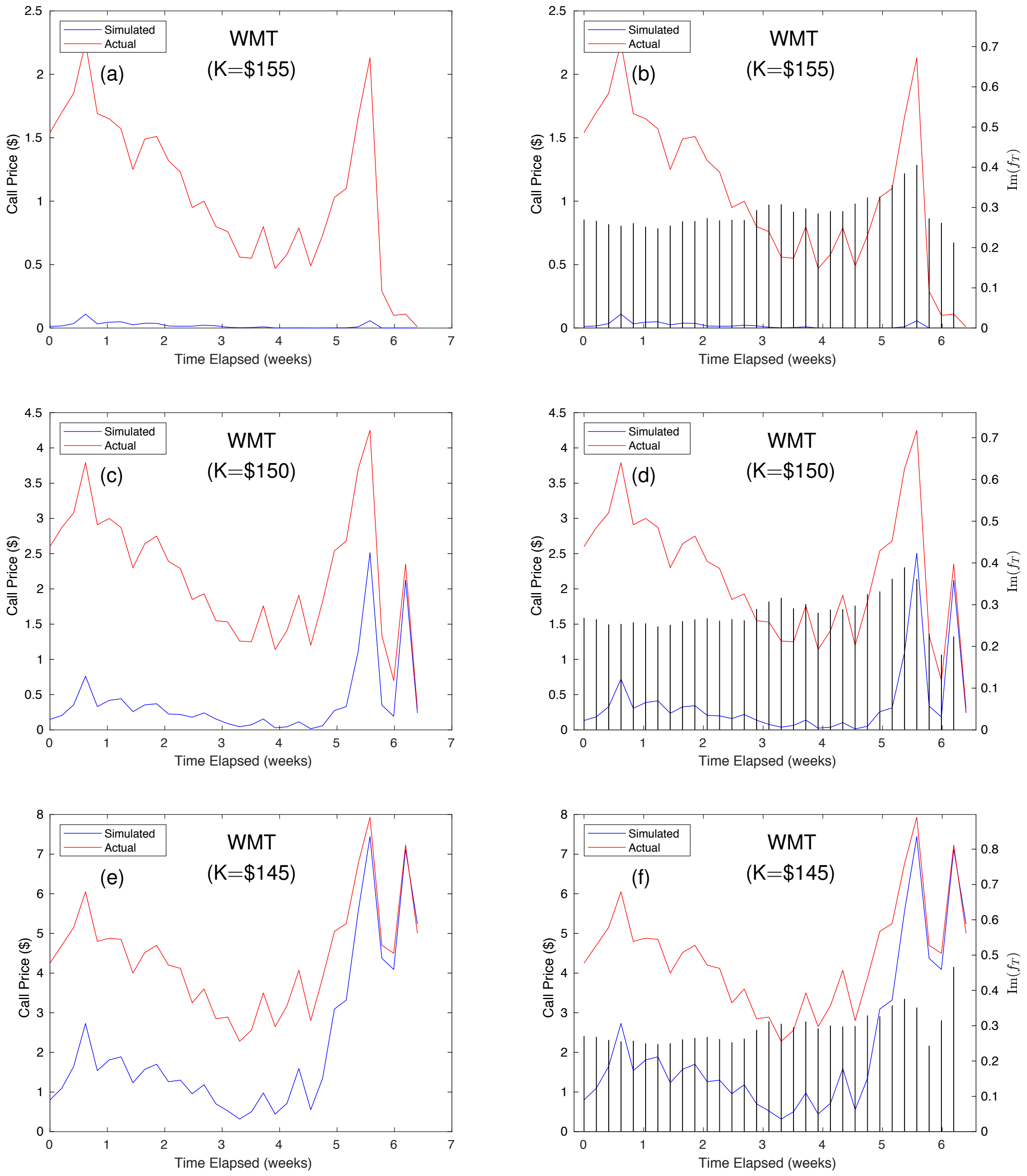

Figure 19. Comparison between actual prices of a European call option (red lines) for stocks of Walmart Inc. and (a,c,e) the original Black–Scholes model based on the pricing formulae (2) and (3) (blue lines), and (b,c,d) the expanded Black–Scholes model based on the pricing formulae (4) and (5) of the expanded Black–Scholes model proposed by Segal and Segal (black vertical lines).

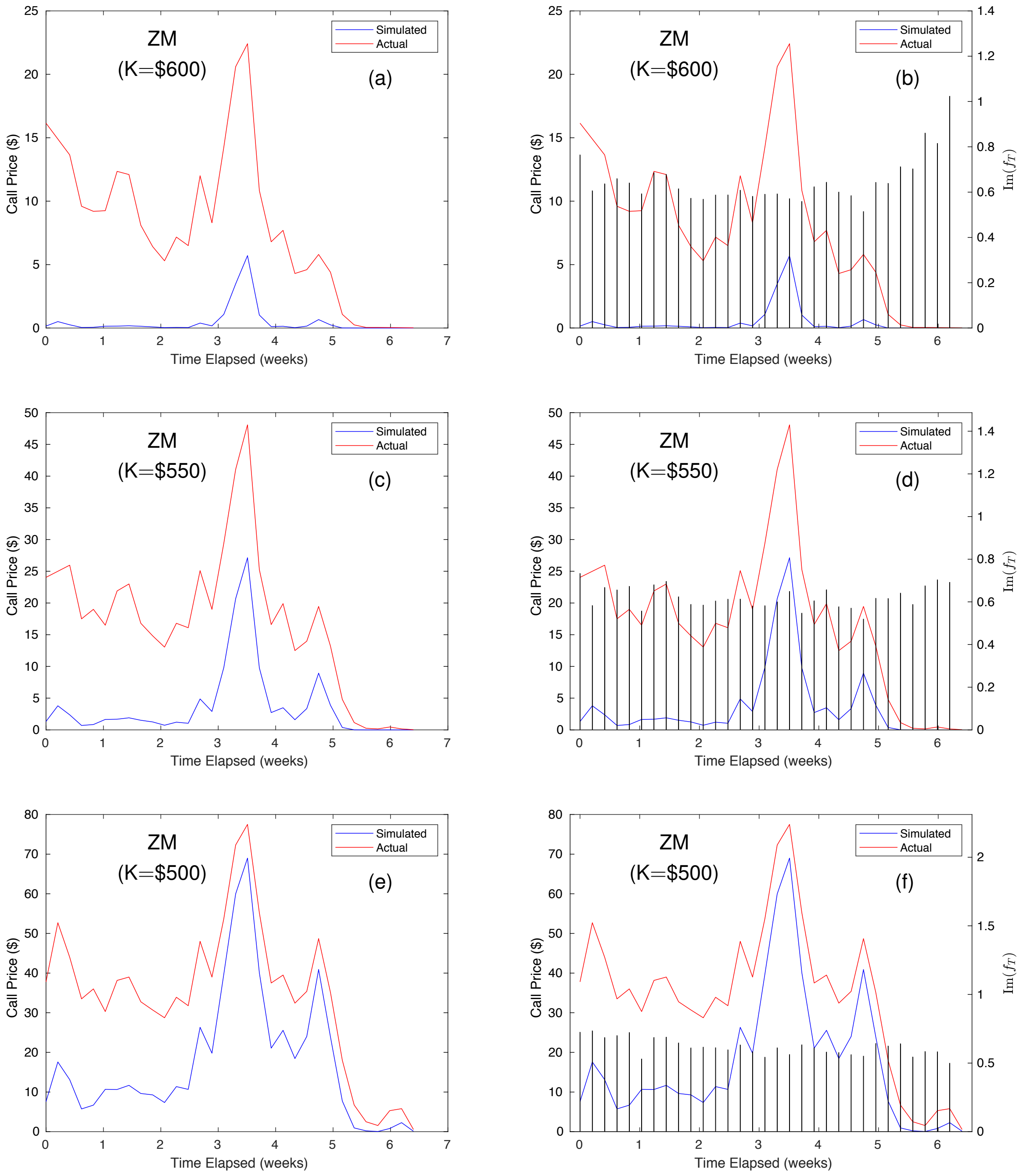

Figure 20. Comparison between actual prices of a European call option (red lines) for stocks of Zoom Video Communications Inc. and (a,c,e) the original Black–Scholes model based on the pricing formulae (2) and (3) (blue lines), and (b,c,d) the expanded Black–Scholes model based on the pricing formulae (4) and (5) of the expanded Black–Scholes model proposed by Segal and Segal (black vertical lines).
